# Response to Androgens and Androgen Receptor Antagonists in the Presence of Cytokines in Prostate Cancer

**DOI:** 10.3390/cancers13122944

**Published:** 2021-06-12

**Authors:** Zoran Culig

**Affiliations:** Experimental Urology, Department of Urology, Medical University of Innsbruck, Anichstrasse 35, A-6020 Innsbruck, Austria; zoran.culig@i-med.ac.at

**Keywords:** prostate cancer, interleukins, androgen receptor, STAT3, anti-androgens

## Abstract

**Simple Summary:**

Prostate cancer is the most frequently diagnosed non-cutaneous tumor in men in the Western world. Therapy for non-organ confined prostate cancer includes anti-androgens such as bicalutamide, enzalutamide and darolutamide. The androgen receptor is expressed during tumor initiation and progression. Androgen receptor could be activated by interleukins, which are produced by blood cells and adjacent stroma. These cytokines may affect response of tumor cells to anti-androgenic drugs, which are commonly used in prostate cancer therapy. There are several experimental studies showing an effect of anti-cytokine therapies in prostate cancer. However, the clinical translation is limited and more clinical trials are needed to improve action of anti-androgens in prostate cells which are stimulated by cytokines.

**Abstract:**

Non-steroidal anti-androgens have a major role in the treatment of non-localized prostate cancer. Interleukins are involved in the regulation of many cellular functions in prostate cancer and also modify cellular response to anti-androgens. A specific role of selected IL is presented in this review. IL-8 is a cytokine expressed in prostate cancer tissue and microenvironment and promotes proliferation and androgen receptor-mediated transcription. In contrast, IL-1 displays negative effects on expression of androgen receptor and its target genes. A subgroup of prostate cancers show neuroendocrine differentiation, which may be in part stimulated by androgen ablation. A similar effect was observed after treatment of cells with IL-10. Another cytokine which is implicated in regulation of androgenic response is IL-23, secreted by myeloid cells. Most studies on androgens and IL were carried out with IL-6, which acts through the signal transducer and activator of the transcription (STAT) factor pathway. IL-6 is implicated in resistance to enzalutamide. Activation of the STAT-3 pathway is associated with increased cellular stemness. IL-6 activation of the androgen receptor in some prostate cancers is associated with increased growth in vitro and in vivo. Molecules such as galiellalactone or niclosamide have an inhibitory effect on both androgen receptor and STAT-3 pathways.

## 1. Introduction

Nonsteroidal anti-androgens bind to the androgen receptor (AR) with low affinity and prevent androgen-induced receptor activation. The development of compounds which block AR activation in prostate cancer is a continuous process that has led to improvements in clinical care. Several decades ago, medical intervention in prostate cancer was based on the application of the progestagenic drug cyproterone acetate. Treatment of cancer tissues with anti-androgens may lead to occurrence of point mutations which change agonistic/antagonistic properties of drugs. The agonistic effects of anti-androgens, however, do not allow individualized treatment because routine investigations to detect a mutation are not a part of clinical practice. The heterogenous nature of prostate cancer means that different AR may be detected in the tissue of a single patient. Further development of nonsteroidal anti-androgens in prostate cancer became possible after the successful use of enzalutamide in clinics. There are currently more efforts to intervene in patients who do not respond to enzalutamide. For this purpose, preclinical and clinical studies with darolutamide have been conducted. A larger number of available anti-androgens is beneficial in precision medicine, in particular because of the fact that there are multiple resistance mechanisms. Th replacement of an anti-androgen in cancer therapy with a different drug could be therefore justified in several cases.

The effectiveness of anti-androgens on inhibition of tumor growth in vitro and in vivo is also determined by other factors, such as coactivators, which interact with one or more domains of the receptor. Importantly, the expression of cytokines, which act in an autocrine or paracrine manner in prostate cancer, has received considerable attention over the last three decades. In prostate cancer, the effects of interleukins (IL)- 8-6, and -1 have been investigated in multiple in vitro and in vivo models. These cytokines are in part implicated in androgenic regulation and the interaction with anti-androgens will be discussed in more detail in this review. Most effects of cytokines on modulation of response to anti-androgens have been detected in cell lines, which represent late stages tumors. In terms of appropriate models, it is challenging to investigate interactions between interleukins and AR in cancer precursor lesions, such as high grade prostate epithelial neoplasia. There is a limited number of appropriate models for prostate interaepithelial neoplasia.

The main aim of this review is to summarize the effects of interleukins at different stages of prostate cancer with emphasis on interactions with androgens and AR. Most papers selected in this review were published after 2015. Some key manuscripts in the field published before that year have also been included. Research with multiple cellular and animal models with clinical implications has received particular attention.

## 2. Multiple Effects of Interleukin-8 in Prostate Cancer

Nuclear factor kappa B, which is highly expressed in prostate cancer, is a major regulator of interleukin expression. It is also a key factor in the up-regulation of IL-8 in benign and malignant tissues. IL-8 (CXCL8) is a chemokine produced by endothelial cells, monocytes, other stromal cells, and epithelial cells. It transmits signals through G protein-coupled receptors CXCR1 and CXCR2. IL-8 is involved in the regulation of prostate vasculature and apoptosis [1]. High expression of IL-8 was observed in areas of prostate inflammatory atrophy and in high grade cancer areas. It was shown that there is an inverse correlation between the expression of IL-8 and AR in prostate cancer tissue. This could be explained by repression of the IL-8 promoter by androgens. Interestingly, there was no difference in IL-8 expression between the races. Investigation of racial differences in oncogene expression in prostate cancer is important because the prognosis is in general bad in African Americans. IL-8 is a cytokine which is highly expressed in the microenvironment of aggressive tumors. Other researchers have investigated the possible effects of IL-8 on AR. It was shown that AR expression and function increase after treatment with IL-8 [2]. Blockade of CXCL2 abolished the effects of IL-8 on AR-mediated transcription. IL-8 up-regulated classic AR target genes, such as cyclin-dependent kinase 2 and prostate-specific antigen. In cells in which IL-8 is overexpressed, effectiveness of bicalutamide was reduced. Stable expression of IL-8 in prostate cancer cells accelerated growth of LNCaP xenografts. In those experiments, growth was associated with increased microvessel density [3]. IL-8 itself could also up-regulate nuclear factor kappa B in cancer cells, thus contributing to expression of other cytokines involved in cancer development. The mechanism of IL-8-induced regulation includes regulation of cyclin D [4].

In prostate cancer, there is an increased interest in the function of neuroendocrine cells. They may have a more important role in late tumor stages than previously anticipated. IL-8 may have an important role also in regulation of the neuroendocrine phenotype in prostate cancer. Neuroendocrine cells express on their surface the receptor CXCR2. Its expression increases in prostate cancer cells with a higher Gleason score. Those cells could also be detected in a tumor microenvironment. The relationship between the role of IL-8 and tumor angiogenesis could be explained by the secretion of proangiogenic factors in neuroendocrine cells. Inhibition of CXCR2 showed an effect in therapy-resistant C4-2 tumors. This approach may be combined with AR blockade in vivo [5]. Although mechanistic studies on IL-8 interaction with AR are available, additional research in the field is needed to improve therapeutic translation and clinical trials designed to inhibit IL-8 and AR signaling.

## 3. Interleukins-1, -10- and -23 Have Different Effects on the Androgen Receptor Pathway

Several other IL may exhibit different effects on AR expression and functional activity. IL-1 is a cytokine expressed mostly in monocytes and has an important role in the regulation of inflammatory response. The p65 subunit of nuclear factor (NF) kappa B is a canonical IL-1 signal transducer. It was found to mediate repression of AR expression and activity [6]. Continuous exposure to IL-1 leads to androgen independence [7]. In prostate cancer, cell sublines established after chronic exposure to IL-1 high levels of human glandular kallikrein and the tumor suppressor NKX 3.1 were measured. IL-1 was demonstrated to inhibit AR and its target genes such as prostate-specific antigen in LNCaP cells [8]. This cytokine up-regulates p62, which is important for cellular survival [9]. The cytokine treatment of LNCaP cells yielded a gene expression pattern similar to that of AR-negative cells [10]. In particular, genes responsible for inflammatory response were found to be expressed at a higher level. So far, there are no successful therapeutic approaches which could target IL-1 in castration therapy-resistant prostate cancer.

IL-10 is an inhibitor of tumor immune response and has a direct negative effect on the expression of NF kappa B. It is expressed mostly in monocytes and Th2 lymphocytes. IL-10 has been identified as one of the factors which regulates neuroendocrine differentiation [11]. Neuroendocrine tumor is an aggressive form of prostate cancer for which anti-androgens cannot be administered. Interestingly, in the same study, a similar effect of enzalutamide on neuroendocrine cells was observed. The results of the study thus imply that inhibition of AR function leads to increased neuroendocrine phenotype and novel experimental strategies should be discussed in order to improve prostate cancer treatment. AR activity was inhibited by IL-10 in prostate cancer cells. Interestingly, IL-10 increased expression of PDL1, which promotes tumor cell survival by interaction with PD1, which is an inhibitory receptor for immune cells. Immunotherapies for prostate cancer are a focus of scientific investigation. A vaccine, Sipuleucel, has been approved by the FDA. For this reason, anti-IL-10 therapies may improve the outcome of tumor therapy in the future.

IL-23 is a heterodimer which consists of two subunits, IL12B und IL23A. It belongs to the IL-12 group of cytokines. IL-23’s inhibitory approaches have been developed in human medicine to treat psoriasis. IL-23 could inhibit the effects of the anti-androgens enzalutamide and darolutamide on induction of cellular senescence. IL-23 was found to activate AR-mediated transcription [12]. High IL-23 levels were measured in sera from patients with advanced prostate cancer. It is secreted by myeloid-derived suppressor cells, which are increasingly present in the prostate tumor microenvironment [13]. The results obtained with human and mice prostate cancer cells lines were validated in organoid cultures. Thus, IL-23 acts as a cytokine which promotes castration therapy resistance. In experiments in which the effect of IL-23 was abolished, prostate cancer cells could become more sensitive to enzalutamide. The effect of IL-23 was also demonstrated in vivo. Activation of IL-23 leads to stimulation of the retinoic acid receptor-related orphan receptor gamma pathway and also activation of signal transducer and activator of transcription (STAT) factor-3. Similarly as in case of CXCR2, targeting IL-23 may increase efficacy of the enzalutamide treatment. Taken together, cytokines affect AR-mediated transcription in different ways, ranging from stimulation of receptor activity to down-regulation of the AR-mediated signaling. In the future, targeting of IL-23 may be confirmed as a valid immunotherapy strategy in prostate cancer.

## 4. Expression and Function of Interleukin-6 and Respective Signaling Pathways in Prostate Cancer

For several reasons, IL-6 is one of the most frequently investigated cytokines in prostate cancer. Since IL-6 and its receptor are expressed in prostate cancer tissue and in the microenvironment, the effects of the cytokine were investigated in cellular and animal models. In general, IL-6 is considered a pleiotropic cytokine. There may be different reasons for these multifunctional effects of IL-6 but it should be kept in mind that signaling pathways of STAT-3, mitogen-activated protein kinase, and phosphatidylinositol 3-kinase may be activated in various cell types. Each of these pathways is regulated by endogenous inhibitors, which are expressed at different levels. Most studies were focused on the expression and phosphorylation of STAT-3 in various prostate cancer models. IL-6 is also considered a valid target and has received a considerable attention in pre-clinical and clinical experimental therapy studies. At this stage, there is no consensus regarding use of clinical anti-IL-6 therapy in patients with prostate cancer.

One of the reasons for scientific interest in IL-6 in prostate cancer is its involvement in cellular plasticity. It is important to understand regulation of epithelial to mesenchymal transition and cellular stemness by IL-6. In conditions in which AR expression decreases, IL-6 expression may increase, thus leading to increased phosphorylation of STAT-3 and appearance of cancer stem cells [14]. These stem cells are of particular interest in prostate cancer because of the lack of therapy options that could prevent the appearance of these cells. IL-6 up-regulation, which leads to increased cellular stemness, may be the consequence of a high reactive oxygen species status [15]. It has been shown that IL-6 and activated STAT-3 lead to increased formation of tumor spheres, which initiated tumor growth in vivo. IL-6 expression is also up-regulated by early growth response 3, which is in part influenced by the NF kappa B pathway [16]. The IL-6 promoter contains early growth response consensus binding sites. The role of IL-6 has been frequently studied in prostate cancer progression. The activation of macrophages by high mobility group box 1 leads to increased expression of IL-6, which is one of the inducers of neuroendocrine differentiation in the prostate [17]. Activated STAT-3 is needed for the expression and transcription of high mobility group box 1. It was proposed that therapies for prostate cancer may include blockade of high mobility group box 1 and the IL-6 receptor. In this way, therapeutic response to enzalutamide may be improved. The effects of tumor-associated macrophages on neuroendocrine phenotype were observed in prostate cancer xenografts. STAT-3 activation could also be achieved by other growth factors, which include transforming growth factor beta. Metformin is an anti-diabetic drug, which is also of potential therapeutic interest in prostate cancer. It was reported that targeting STAT-3 by metformin enhances therapeutic response to enzalutamide [18]. Metformin may exert its effect in part by inhibiting epithelial to mesenchymal transition.

## 5. Possibility for Simultaneous Targeting of Androgen Receptor and STAT-3 in Prostate Cancer

An important area of research in prostate cancer is the interaction between signaling pathways of IL-6 and AR. Different approaches were developed with the aim to target both pathways in prostate cancer. STAT-3 inhibition by a small molecule AG490 could lead to reversal of enzalutamide resistance [19]. On the other hand, overexpression of constitutively active STAT-3 in prostate cancer cells contributes to enzalutamide resistance. Mast cells may enhance AR activity by cytokine signaling [20]. In those experiments, it was shown that additional experimental treatment with an inhibitor of miRNA32 is necessary to prevent enzalutamide induction of neuroendocrine phenotype. In addition to enzalutamide, AR degraders may be available for therapy. In this context, it was proposed that enzalutamide may be different to other anti-androgens such as the AR degrader ASC-J9 in modulation of the CCL2/STAT-3 response [21]. Ligand-independent and synergistic activation of the AR by IL-6 is an important mechanism that contributes to prostate cancer progression. IL-6 is known as a molecule which reduces concentration of androgen needed for maximal activation of the AR [22,23]. This effect of IL-6 on proliferation and other cellular events may depend on a cellular context. Thus, different effects in LNCaP or MDA PCa 2b cells were described [22,23]. Inhibition of LNCaP cellular proliferation by IL-6 is in contrast to the growth-promoting effect, which was observed in MDA PCa 2b cells in vitro and in vivo. Differences in function of AR coactivators in these two models may account for contrasting effects in growth regulation. Another anti-androgen, which may at least in part block AR activation by IL-6, is bicalutamide. However, IL-6 may up-regulate the coactivator TIF2 in order to diminish the effect of bicalutamide on AR-mediated transcription [24].

The activation of IL-6 signaling pathways is influenced by other proteins, such as tyrosine kinase Fer, which phosphorylates AR at tyrosine 223 and binds the receptor through the SH2 domain [25]. Another kinase that is important for phosphorylation of the AR is PIM [26]. Phosphorylation of the AR was reduced in the presence of PIM inhibitors. Several AR phosphorylation sites are present in the N-terminal region of the receptor. These findings have a clinical significance. High levels of phosphorylated PIM1 at S213 were observed in clinically aggressive prostate cancer. There are other examples that show how signaling pathways of IL-6 interact with those of other growth factors. IL-6 signaling could be linked to those of the pathway insulin-like growth factor I [27]. Insulin-like growth factors belong to the main anti-apoptotic regulators in the prostate. Many prostate cancers do not express the tumor suppressor PTEN. FOXO1 is a PTEN downstream factor, which also prevents interaction between IL-6 and AR [28]. Down-regulation of FOXO1 may thus lead to enhanced AR activation by androgen and IL-6.

Among different approaches to target AR and STAT-3 pathways, administration of niclosamide should be mentioned [29]. Niclosamide was initially discovered as an inhibitor of a constitutively active AR (V7). Niclosamide inhibited cell growth, colony formation, cellular migration and invasion, and induced apoptosis.

Action of IL-6 in prostate cancer may also be supported by other cytokines, such as oncostatin. IL-6 and oncostatin synergize with the signaling pathway of phospahtydilinositol 3-kinase, which is associated with the failure of endocrine therapy [30].

IL-6 not only regulates activity of the AR but is also important for regulation of androgen synthesis [31]. IL-6-mediated up-regulation of androgen synthesis is in part mediated by a high expression of the steroidogenic anzyme AKR1C3. Higher testosterone levels were detected in tumors generated from IL-6-overexpressing cells, which were orthotopically inoculated into the prostate of castrated mice. These results provide an interesting link between cytokine treatment and intracrine synthesis of androgens in prostate cancer.

Another element of the interleukin-6 signaling pathway, which influences response to androgens and enzalutamide, is suppression of cytokine signaling (SOCS)-3 [32]. SOCS normally prevents continuous activation of the signaling pathway of STAT-3. Enzalutamide and SOCS-3 could inhibit androgen receptor activity. Hypermethylation of SOCS-3, which is frequently seen in prostate cancer tissues, could lead to a reduced efficacy of enzalutamide. Inhibition of multiple pathways, including those of AR and STAT-3 in prostate cancer, could be achieved by treatment with galiellalactone [33]. Galiellalactone is a small molecule that inhibits primarily STAT-3. Galiellalactone also decreased the expression of AR-target genes in explants from benign and malignant prostate models, thus confirming the importance of inhibition of multiple signaling pathways in clinical prostate cancer. STAT-3 is also expressed and phosphorylated in metastatic lesions of prostate cancer [34].

There also are other endogenous inhibitors of expression of IL-6 in prostate cancer, such as Sprouty2 [35]. Sprouty2 is a tumor suppressor frequently deleted in prostate cancer. IL-6, which is as a consequence up-regulated, elevates the levels of circulating cholesterol by lipolysis and hepatic cholesterol biosynthesis. Cholesterol uptake is increased through scavenger receptor type B.

The findings described above for prostate cancer are also of importance in other human tumors. Interaction between interleukin-6 and AR was also described in esophagus cancer, where the presence of AR is associated with a bad prognosis [36]. In bladder cancer cell lines, inhibition of AR activity by enzalutamide also down-regulates IL-6 expression [37]. Molecules implicated in cancer progression such as matrix metalloproteinase-2 were also inhibited.

In prostate cancer, it is important to analyze IL-6 signaling in the context of coexpression with ERG. ERG is a member of the E-26 transformation-specific (ETS) family of transcription factors, which regulate multiple cellular functions. There is a considerable percentage of prostate cancers in which TMPRSS: ERG fusion was detected. IL-6 is expressed in ERG-positive cancers in which its up-regulation is caused by prostaglandin receptor E2 [38].

Finally, it is important to state that the presence of the AR is not always necessary for the effects of cytokine and chemokines [39]. It was described that in fibroblasts which are adjacent to tumor tissue, AR expression may decrease, thus leading to increased expression of IL-8 and the CC-chemokine ligand 2 (CCL2) and stimulation of migration and invasion. Thus, these chemokines may be particularly active in conditions in which AR signaling is limited.

For anti-IL-6 clinical trials in prostate cancer, it is important to appropriately approach individual patients. Responsiveness to therapy with the anti-IL-6 antibody such as siltuximab may depend on the general status of a patient, previous therapies, and expression of regulators of cytokine signaling.

## 6. Conclusions and Future Perspectives

Antiandrogens frequently used in prostate cancer and the respective mechanisms of resistance are mentioned in Table 1. The sources and role of most important cytokines in prostate cancer is summarized in Table 2. Although multiple targets have been identified in advanced prostate cancer in experimental studies, much more effort is needed for improvement of clinical therapy. Since one of the reasons for up-regulation of IL-6 in prostate cancer may be androgen ablation, an anti-IL-6 treatment in early stages is an option for discussion. More research is also needed to identify biomarkers that will guide urologists and oncologists in individualized treatment of patients. There are interesting results obtained with niclosamide and galiellalactone, which target both androgen and IL-6 signaling pathways. Research in the last decade also indicates that, in addition to IL-6, there are several other cytokine and chemokines serving as targets in prostate cancer. Because of different interactions with the androgen signaling pathway, combinations with anti-androgens may be a reasonable treatment option in the future. Importantly, any up-regulation of cytokines during androgen ablation or AR blockade should be subjected to a combination therapy in the future. Such therapies may be available if hypothesis-driven clinical research is carried out. Smaller studies will likely lead to multicenter clinical trials, which will evaluate the effectiveness of novel drugs that target cytokine signaling in prostate cancer.

## Figures and Tables

**Table 1:** Frequently used anti-androgens in prostate cancer therapy in the past and present.

Antiandrogen	Structure	Mechanisms of Resistance
Cyproterone acetate	Steroidal	AR mutations
Hydroxyflutamide	Non-steroidal	AR mutations, hypersensitivity, coactivator alterations
Bicalutamide	Non-steroidal	AR mutations
Enzalutamide	Non-steroidal	AR variants, cytokine interaction
Darolutamide	Non-steroidal	AR variants, cytokine interaction

**Table 2:** Summary of interactions between important interleukins and the androgen signaling pathway in prostate cancer.

Interleukin	Source	Oncogene/Tumor Suppressor	Regulation of Androgen Signaling
8	Autocrine/paracrine	Oncogene	Reduces anti-androgen sensitivity
1	paracrine	Tumor suppressor	Inhibits AR
10	paracrine	Oncogene	Inhibits AR
23	paracrine	Oncogene	Activates AR
6	Autocrine/paracrine	Oncogene (in some circumstances tumor suppressor)	Activates AR
